# Light availability affects stream biofilm bacterial community composition and function, but not diversity

**DOI:** 10.1111/1462-2920.12913

**Published:** 2015-07-22

**Authors:** Karoline Wagner, Katharina Besemer, Nancy R. Burns, Tom J. Battin, Mia M. Bengtsson

**Affiliations:** ^1^ Department of Limnology and OceanographyUniversity of ViennaViennaAustria; ^2^ WasserCluster LunzLunz am SeeAustria; ^3^ School of EngineeringUniversity of GlasgowGlasgowUK; ^4^ Stream Biofilm and Ecosystem Research LaboratorySchool of Architecture, Civil and Environmental EngineeringEcole Polytechnique Fédérale LausanneLausanneSwitzerland

## Abstract

Changes in riparian vegetation or water turbidity and browning in streams alter the local light regime with potential implications for stream biofilms and ecosystem functioning. We experimented with biofilms in microcosms grown under a gradient of light intensities (range: 5–152 μmole photons s^−1^ m^−2^) and combined 454‐pyrosequencing and enzymatic activity assays to evaluate the effects of light on biofilm structure and function. We observed a shift in bacterial community composition along the light gradient, whereas there was no apparent change in alpha diversity. Multifunctionality, based on extracellular enzymes, was highest under high light conditions and decoupled from bacterial diversity. Phenol oxidase activity, involved in the degradation of polyphenolic compounds, was twice as high on average under the lowest compared with the highest light condition. This suggests a shift in reliance of microbial heterotrophs on biofilm phototroph‐derived organic matter under high light availability to more complex organic matter under low light. Furthermore, extracellular enzyme activities correlated with nutrient cycling and community respiration, supporting the link between biofilm structure–function and biogeochemical fluxes in streams. Our findings demonstrate that changes in light availability are likely to have significant impacts on biofilm structure and function, potentially affecting stream ecosystem processes.

## Introduction

Benthic ecosystems are of global significance for biogeochemistry and biodiversity (Covich *et al*., 2004; Findlay and Battin, in press). In streams, the benthic zone is a thin but critical layer that connects surface with subsurface habitats, including the hyporheic zone and groundwater. Key stream ecosystem processes including nutrient cycling, primary production and respiration are linked to the benthic zone. Benthic biofilms dominate the microbial life in streams (Geesey *et al*., 1978) and carry out fundamental ecosystem processes such as primary production and organic matter processing (Romani and Sabater, 1999; Battin *et al*., 2003a; 2008). The study of the algal component in benthic biofilms, traditionally termed periphyton, has been a mainstay in stream ecology over the last decades (e.g. Hill *et al*., 2003). It is well established that the physical structure and composition of algal communities depend on the flow and light regime (Hill and Boston, 1991; Wellnitz and Rader, 2003). Benthic algae and cyanobacteria are the most important primary producers in streams (Lamberti and Steinman, 1997) and form the trophic basis for invertebrate grazers in streams and are therefore key for carbon transfer (Power *et al*., 1985).

The appreciation of the prominent role of heterotrophic bacteria in benthic biofilms for ecological and biogeochemical processes in streams is more recent (Findlay, 2010). Given the close spatial proximity of heterotrophic microorganisms and phototrophs such as algae in biofilms, algal‐bacterial interactions have received some attention (e.g., Romani and Sabater, 1999; Rier and Stevenson, 2002; Ylla *et al*., 2009; Rier *et al*., 2014). It has been shown, for instance, that algal–bacterial interactions are pronounced when algal exudates are abundant during periods of high light availability and photosynthesis, but low when external substrates satisfy the heterotrophic carbon demand in biofilms (Rier and Stevenson, 2002; Ylla *et al*., 2009). It has also been purported that the interaction between algae and microbial heterotrophs in biofilms could induce priming of putatively recalcitrant dissolved organic matter (DOM) from the terrestrial milieu (Bengtsson *et al*., 2014; Rier *et al*., 2014); evidence remains weak, however. Although algal–bacterial interaction in benthic biofilms are likely critical for carbon fluxes in streams (Rier and Stevenson, 2002; Ylla *et al*., 2009), there is little information on how bacterial community composition and function respond to variation in primary productivity, for example due to varying light availability in streams.

The natural light regime in streams is increasingly becoming perturbed. For instance, the riparian deforestation causes high levels of photosynthetically active radiation (PAR) and ultraviolet radiation to reach the streambed, with consequences for algal biomass, primary production and nutrient cycling (e.g. Sweeney *et al*., 2004; Richardson and Béraud, 2014). Furthermore, ‘browning’ as induced not only by increased terrestrial deliveries of humics, but also elevated turbidity because of increasing erosion may attenuate light intensity in freshwater ecosystems (Karlsson *et al*., 2009). Finally, emerging nighttime light pollution may increasingly impact stream ecosystems (Perkin *et al*., 2014). It is therefore critical to understand the effect of light on microbial biofilms in streams beyond the mere effects that light has on algae.

In this study we experimented with benthic biofilms in microcosms under a gradient of six different light intensities to establish how light impacts bacterial community composition, diversity and community function. While it has been established that light intensity affects algal biomass and activity in biofilms (Adlboller, 2013; Ceola *et al*., 2013), we hypothesized that light also affects bacterial community composition as modulated by phototrophic biomass and primary productivity. This is based on the observation that microbial heterotrophs in biofilms rely more on allochthonous carbon sources (i.e. terrestrially derived DOM) if supply from biofilm phototrophs is reduced (Battin *et al*., 2003b; Ylla *et al*., 2009), which potentially selects for a different bacterial community. At intermediate light intensities, we hypothesized that biofilms may rely to a similar extent on autochthonous DOM sources (i.e. from biofilm phototrophs) and allochthonous DOM sources. Therefore, we expected to observe a peak in bacterial alpha diversity (richness and evenness of operational taxonomic units, OTUs) under these circumstances due to increased resource diversity. We further anticipated a shift in community function along the light gradient, reflecting the decreased reliance of bacteria on phototroph exudates under low light availability. We used 454‐pyrosequencing of the 16S rRNA gene to address biofilm bacterial community composition and diversity in combination with extracellular enzyme activity assays to study community function. Furthermore, a multifunctionality index was calculated from the individual enzyme activities to study the effects of biodiversity on multiple ecosystem functions, which is important to avoid the overestimation of functional redundancy in a given ecosystem (Gamfeldt *et al*., 2008; Peter *et al*., 2011). Our focus on bacterial community composition, biodiversity and multifunctionality expands existing knowledge on the relationship between light and biofilms (e.g. Rier and Stevenson, 2002; Lear *et al*., 2008; Rier *et al*., 2014) and sheds new light on community structure and function in stream biofilms.

## Results

### Influence of light on biofilm biomass and activity

**Figure 1:**
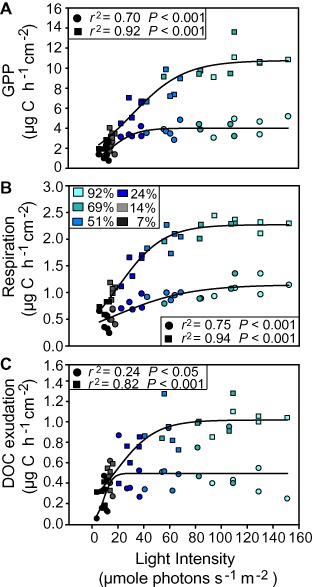
Non‐linear (hyperbolic) regression between GPP (A) respiration (B) and DOC exudation (C), respectively, and the measured light intensities for each microcosm at day 1 (circles) and at day 7 (squares) of the experiment; same colours indicate light treatments (relative transmission (%T) of the incident light).

We grew benthic biofilms over a total of 27 days in stream‐side flumes under 6 different light intensities (generated by shading foils) with 92%, 69%, 51%, 24%, 14% and 7% transmission of the incident light (henceforward termed 92%T, 69%T, 51%T, 24%T, 14%T and 7%T light treatment respectively). This yielded mature biofilms with a biomass ranging from 0.04 to 0.51 mg C cm^−2^ that we gently transferred into laboratory microcosms covered with the same shading foils as during the initial growth phase. Higher light generally resulted in an increase in biofilm biomass, chlorophyll *a* content and bacterial cell counts (first measured on day 1 directly after the transfer to the microcosms), yet the highest values were found at intermediate light intensities (Table S1); this increase was more pronounced after 7 days of experimental work in the microcosms (day 7). These time points were chosen to be representative of the experimental period, representing conditions shortly after the transfer of the biofilms from the field (day 1) and after acclimatization to laboratory conditions (day 7). Inevitably, the laboratory conditions differed from the field conditions, which in part explains the differences observed between time points for most measured parameters (see SI methods for a summary). These temporal changes are not the focus of this study, instead effects attributable to the light gradient observed for both time points are henceforth reported. The light gradient yielded biofilms with varying phototrophic biomass, which resulted in a clear gradient of primary productivity (day 1: *r* ^2^ = 0.70, *P* < 0.001, day 7: *r* ^2^ = 0.92, *P* < 0.001), with saturating relationships for gross primary production (GPP) (Fig. 1A). Also, community respiration (R) significantly increased along the light gradient (day 1: *r* ^2^ = 0.75, *P* < 0.001, day 7: *r* ^2^ = 0.94, *P* < 0.001) (Fig. 1B). Net primary production (NPP) was always positive throughout the experiment, even under low light availability (Table S1). A ratio of NPP to R of 2.0 ± 0.7 (mean ± SD) in the 7%T light treatment indicates high primary productivity and net‐autotrophy even under low light conditions. There was also a net release of dissolved organic carbon (DOC) from the biofilms, which significantly increased with light availability (day 1: *r* ^2^ = 0.24, *P* < 0.05, day 7: *r* ^2^ = 0.82, *P* < 0.001) and probably reflects exudation by biofilm phototrophs (Fig. 1C). However, the presumed simultaneous uptake of DOC by heterotrophs could not be quantified in this study and likely conceals some of the DOC exuded by phototrophs.

### Influence of light on biofilm community composition, diversity and multifunctionality

**Figure 2:**
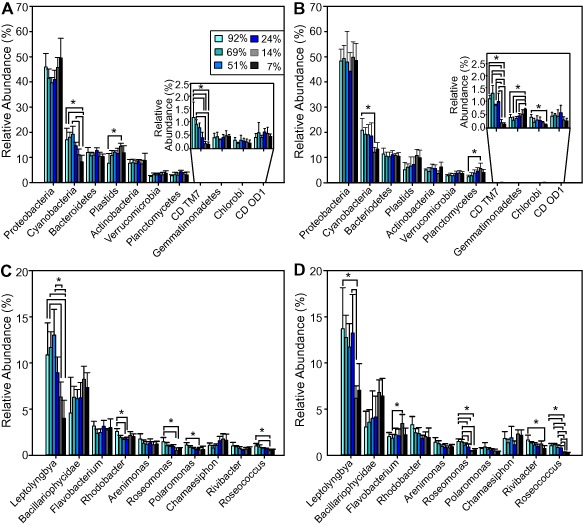
Relative abundance of taxa based on 454‐pyrosequenicng of the 16S rRNA gene at the phylum level at day 1 (A) and at day 7 (B) of the experiment and at the genus level at day 1 (C) and at day 7 (D) of the experiment. The most abundant taxa are displayed on each level. Results are based on taxonomic classification of 97% OTUs. An asterisk (*) indicates significant differences (ANOVA, *P* < 0.05) between light treatments (relative transmission (%T) of the incident light) connected with brackets. Error bars indicate ± 1 standard deviation of the mean based on five replicate samples.

454‐pyrosequencing of the 16S rRNA gene and taxonomic classification using classification resources for environmental sequence tags (CREST; Lanzén *et al*., 2012) revealed clear effects of light on the relative abundance of the phototrophic taxa of the biofilms. The effect of light was particularly evident for *Cyanobacteria*, which showed higher relative abundance under high light conditions both at day 1 and at day 7 of the experiment (Fig. 2A and B). *Cyanobacteria* were the second most abundant taxon and showed significant increases with GPP (*r_s_* = 0.72, *P* < 0.001), whereas plastids showed a significantly decreasing relationship with GPP (*r_s_* = −0.60, *P* < 0.001). Impacts of the light gradient on the bacterial community were evident on both phylum and genus levels (Fig. 2). Taxa that showed a higher relative abundance under high light conditions included the alphaproteobacterial genera *Roseomonas*, *Rhodobacter* and *Roseococcus*, the betaproteobacterial genera *Polaromonas* and *Rivibacter* (Fig. 2C and D), as well as unidentified taxa belonging to the candidate division TM7. The opposite trend was found within the *Planctomycetes* and *Gemmatimonadetes* (Fig. 2A and B), which were more abundant under low light conditions at day 7. Biofilm community composition also shifted from day 1 to day 7 of the experiment. The relative abundance of the betaproteobacterial genus *Rivibacter* increased significantly during the experiment, while the relative abundance of *Bacillariophyta* (Plastids) and the genus *Flavobacterium* (Bacteroidetes) decreased (Fig. 2).

**Figure 3:**
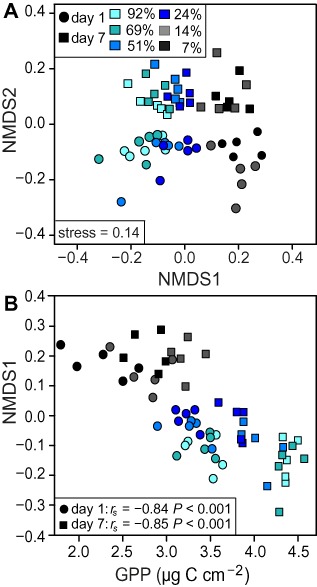
Non‐metric multidimensional scaling (NMDS) ordination based on Bray–Curtis distance of the bacterial community composition (97% OTU relative abundances) (A). Spearman's rank correlation between the first axis of the NMDS ordination and GPP (log transformed) (B). Colours indicate light treatments (relative transmission (%T) of the incident light).

To address the indirect effect of light on bacterial community composition modulated by phototrophic activity we performed non‐metric multidimensional scaling (nMDS) ordination excluding OTUs clustered at a 97% identity level (97% OTUs) identified as *Cyanobacteria* and algal plastids. The nMDS ordination showed an apparent gradient in community composition in response to light and a clear separation of the bacterial community composition from day 1 to day 7 of the experiment (Fig. 3A). Light (measured light intensities) explained 7% (PERMANOVA: *R* ^2^ = 0.07, *P* < 0.01), and time explained 11% of the variance in community composition (PERMANOVA: *R* ^2^ = 0.11, *P* < 0.01). GPP, a proxy for algal biomass and activity, explained 10% of the variance in community composition (PERMANOVA: *R* ^2^ = 0.10, *P* < 0.01). GPP was also a significant predictor for bacterial community composition when both time points were tested separately (Fig. 3A) and showed a significant correlation with the first axis of the nMDS ordination (Fig. 3B).

Richness, evenness, the Simpson as well as the Shannon number equivalents (calculated based on 97% OTU data) were highest in the 24%T light treatment at day 1 of the experiment (Table S2). However, at day 7 of the experiment no significant differences could be observed in any of the alpha diversity measures. No correlation could be detected between richness (day 1: *r_s_* = 0.13 *P* > 0.05; day 7: *r_s_* = −0.11 *P* > 0.05) and evenness (day 1: *r_s_* = 0.17 *P* > 0.05; day 7: *r_s_* = −0.03 *P* > 0.05), respectively, and GPP at day 1 and day 7 of the experiment. Also community respiration showed no significant correlation with species richness (day 1: *r_s_* = −0.12, *P* > 0.05, day 7: *r_s_* = −0.21, *P* > 0.05 of the experiment).

A multifunctionality index was calculated from the area‐specific extracellular enzymatic activities according to Gamfeldt *et al*. (2008). The probabilities to sustain multifunctionality (multifunctionality index) were significantly higher in the 92%T and the 51%T light treatments than in the 7%T light treatment at day 1 and at day 7 of the experiment (Fig. 5A and B). Interestingly, multifunctionality increased in the 92%T and the 7%T light treatments, while it decreased in the 51%T light treatment from day 1 to day 7 of the experiment. There was no significant correlation between multifunctionality and 97% OTU richness neither at day 1 nor at day 7 (day 1: *r_s_* = 0.17 *P* > 0.05, day 7: *r_s_* = 0.17 *P* > 0.05).

### Influence of light on extracellular enzyme activities, DOC and nutrient dynamics

**Figure 4:**
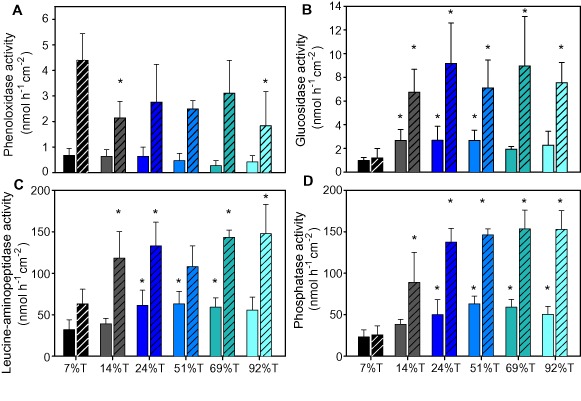
The areal activity rates of the extracellular enzymes phenoloxidase (A), beta‐glucosidase (B), leucine‐aminopeptidase (C) and phosphatase (D). Solid bars indicate results from day 1, and shaded bars from day 7 of the experiment. Error bars indicate 1 standard deviation of the mean based on 5 replicate samples. An asterisk (*) above the bars indicates a significant difference (ANOVA, *P* < 0.05) between the 7% light transmission treatment (7%T, lowest light treatment) and the respective treatment within each time point.

Phosphatase, leucine‐aminopeptidase and beta‐glucosidase activity generally increased with light intensity, whereas phenol oxidase activity decreased from the highest to the lowest light treatment at day 7 (Fig. 4). Beta‐glucosidase activity significantly correlated with community respiration, showing an increase with light availability, whereas phenol oxidase activity showed a decreasing relationship with community respiration (Fig. 6A and B). Phosphatase activity significantly correlated with bulk PO_4_ uptake and leucine‐aminopeptidase activity correlated with NO_3_ uptake from the water (Fig. 6C and D). Mantel's test showed a significant correlation between the extracellular enzyme activities and community composition (day 1: *r* = 0.52, *P* < 0.001, day 7: *r* = 0.44, *P* < 0.001).

**Figure 5:**
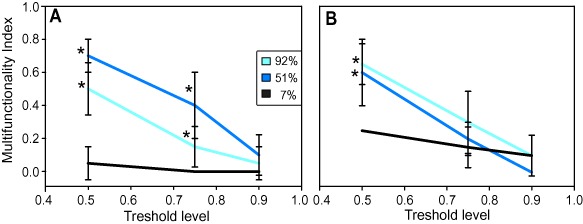
Probabilities that microbial communities of the treatments (selected to improve clarity of presentation) with 92%, 51% and 7% transmission (%T) of the incident light sustain multifunctionality under different threshold levels at day 1 (A) and at day 7 (B) of the experiment. An asterisk (*) indicates significant differences (ANOVA, *P* < 0.05) between the 7%T and the 92%T and 51%T light treatments respectively.

**Figure 6:**
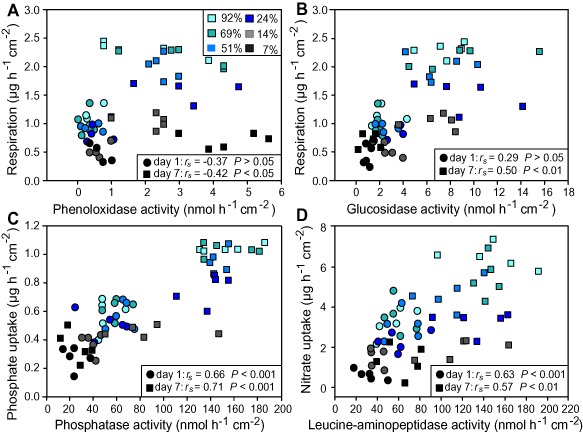
Spearman's rank correlations of extracellular enzyme activities involved in carbon cycling with respiration (A and B) and enzyme activities involved in P‐ and N‐cycling with PO_4_ uptake (C) and NO_3_ uptake (D) at day 1 (circles) and at day 7 (squares) of the experiment. Colours indicate light treatments (relative transmission (%T) of the incident light).

## Discussion

### Biofilm community composition, diversity and multifunctionality

We observed a clear shift in bacterial community composition along the light gradient. This supports our hypothesis that light not only affects phototrophic biomass and activity, but also bacterial community composition. There was a direct effect of light on the phototrophic components of the biofilms such as *Cyanobacteria* and on other likely phototrophic bacterial taxa such as *Rhodobacter* and *Roseococcus* (Yurkov, 2006). In addition to these direct effects, some bacterial taxa with predominantly heterotrophic lifestyles appeared to increase with light (e.g. Candidate division TM7, *Roseomonas* and *Rivibacter*), whereas others decreased (e.g. *Planctomycetes* and *Gemmatimonadetes*). This may reflect preferences towards utilization of autochthonous and allochthonous organic matter, respectively, although it is difficult to make functional predictions based on such trends alone. However, the ordination based on 97% OTUs (excluding Cyanobacterial and plastid OTUs) shows a distinct shift along the light gradient which indicates that the community shift is driven by changes at 97% OTU level, and not changes on phylum or genus level. We chose to not address algal community composition in this study, as pervious work on biofilms from the same watershed showed that light primarily affected algal biomass, not species composition (Adlboller, 2013). It is, however, possible that a shift in the algal community could have contributed to the bacterial community shift due to specific associations between algae and bacteria.

While light availability had a clear influence on bacterial community composition, alpha diversity (e.g. 97% OTU richness and evenness) did not show any obvious trends along the light gradient nor did it correlate with any process (e.g. GPP and community respiration) measured. This is remarkable, because thicker biofilms forming under high light availability could be expected to be structurally more complex, therefore including more niches and ultimately elevated diversity (Jackson *et al*., 2001). Furthermore, according to the classical notion that energy supply limits diversity (Currie, 1991; Cardinale *et al*., 2009), higher primary production under high light conditions may stimulate diversity. However, we observed no relationship between alpha diversity measures of the bacterial community and GPP in biofilms. The lack of a relationship between alpha diversity and community respiration contrasts findings from simple experimentally assembled communities (Bell *et al*., 2005). However, it is consistent with the observation that diversity does not scale with ‘broad’ processes (*sensu* Schimel and Schaeffer, 2012) related to organic matter degradation in soils. Furthermore, it has been proposed that the response of ecosystem processes to increasing microbial diversity saturates as one moves from synthetic assemblages with relatively low diversity to complex and highly diverse natural assemblages (Bell *et al*., 2009). We anticipated that mixing of taxa involved in the degradation of either autochthonous phototroph‐derived DOM or allochthonous DOM would lead to elevated diversity in the treatments with intermediate light availability. Indeed, highest microbial diversity was found in the treatment with 24% transmission of the incident light at day 1 of the experiment, which supports our initial hypothesis that higher resource diversity may lead to elevated microbial diversity. However, this effect was not detectable at day 7 of the experiment, indicative that the community shift from day 1 to day 7 of the experiment or other yet unknown factors were more important.

Multifunctionality can be a useful way to aggregate responses of individual processes particularly if these are ‘narrow’ functions (*sensu* Schimel and Schaeffer, 2012) such as extracellular enzymes expressed to assure a specific physiological pathway or by a phylogenetically constrained microbial group. Our results suggest that multifunctionality as derived from extracellular enzymatic activity was related to light, with highest probability of sustaining multiple enzymatic activities under high light conditions. We propose that the mixture of autochthonous and allochthonous DOM present under high and intermediate light conditions fostered higher community multifunctionality than the allochthonous DOM predominantly available under low light conditions. This finding is consistent with a report on enzyme activity in biofilms showing that resource complexity influenced multifunctionality (Peter *et al*., 2011).

### Phototroph–heterotroph interactions in biofilms

Positive phototroph–heterotroph interactions in biofilms, such as the release of extracellular organic molecules by phototrophs (mainly algae and *Cyanobacteria*) with increasing light intensity (Wood *et al*., 1992) or the supply of a large specific surface area for the attachment of bacteria (Rier and Stevenson, 2002), sustain heterotrophic metabolisms in stream biofilms (Ylla *et al*., 2009). We expected to find enhanced phototroph–heterotroph interactions with increasing light intensity, indicated by a parallel increase in abundance of biofilm phototrophs and heterotrophs with light availability. Indeed, we observed higher bacterial cell counts under high light conditions, which were aligned not only with an increase in biofilm biomass and chlorophyll *a* concentration, but also with ‘broad’ processes such as GPP and DOC exudation, insinuating a positive relationship establishing between biofilm phototrophs and heterotrophs as light intensity increases.

The increased expression of phosphatase, leucine‐aminopeptidase and beta‐glucosidase with light availability indicates enhanced degradation of phototroph exudates, including peptides and simple polysaccharides, and is further evidence for stronger phototroph–heterotroph interactions under high light conditions (Jones and Lock, 1993; Espeland *et al*., 2001; Ylla *et al*., 2009). This corroborates earlier observations on increased expression of leucine‐aminopeptidase and beta‐glucosidase with phototrophic activity in biofilms (Haack and McFeters, 1982; Romani *et al*., 2004). In contrast, under low light availability, biofilms were visibly thinner, developed lower biomass and showed higher phenol oxidase expression than under high light conditions. Phenol oxidase is involved in the degradation of lignin and other polyphenolic compounds (Sinsabaugh, 2010) and might therefore be regarded as an indicator for the degradation of more complex allochthonous DOM constituents. This result contrasts with the notion of ‘priming’, where labile algal exudates stimulate the degradation of allochthonous DOM (Guenet *et al*., 2010). In fact, ‘priming’ would have resulted in higher phenol oxidase activity under high light conditions. Instead, stronger reliance on allochthonous DOM in thin biofilms under low light availability may be attributable to mass transfer phenomena and decreased phototroph–heterotroph interactions. This notion is in fact supported by earlier observations suggesting enhanced mass transfer of DOM from the bulk liquid to and into thin biofilms to satisfy the carbon demand of the microbial heterotrophs (Battin *et al*., 2003b; Romani *et al*., 2004).

Further evidence towards decreased phototroph–heterotroph interactions is provided by increasing ratios of NPP to respiration with light intensity, shifting community metabolism from relatively more heterotrophic under low light conditions to more autotrophic under high light availability. The bacterial community shift from low to high light availability in combination with increased phenol oxidase activity is indicative of a community‐level response to more complex allochthonous carbon sources. Phenol oxidase is assumed to be expressed by a restricted number of microbial taxa (Woo *et al*., 2014). The correlation between community composition and function supports this observation. Altogether, our results are consistent with the view of a biofilm bacterial community that is structured by phototrophs through phototroph–heterotroph interactions under high light, and by allochthonous carbon sources under low light.

### Possible implications for stream ecosystem processes

Our study paves the way towards a better mechanistic understanding of the fine‐scale processes in biofilms that likely affect ecosystem processes as environmental changes such as riparian deforestation, elevated browning or water turbidity alter the light regime in streams. Our findings suggest that light intensity not only affects overall phototrophic biomass and production in benthic biofilms, but also has direct and indirect cascading effects on the biofilm heterotrophs. For instance, we observed correlations between extracellular enzymatic activity, nutrient cycling and community respiration respectively. We do acknowledge that these correlations may be partly driven by biomass accrual along the light gradient. Nevertheless, these patterns also support the notion of a link between biofilm structure–function and biogeochemical fluxes in streams. Under high light availability, phototrophs likely provide heterotrophs with organic nitrogen and phosphorous, while satisfying their own nutrient demand from inorganic nutrients in the streamwater. It is remarkable that despite lower biomass, biofilms grown under low light availability appeared to be more efficient in degrading allochthonous DOM as indicated by the higher phenol oxidase activity. Thus, stream reaches that experience limited light, for example, due to intact riparian vegetation, may process more allochthonous DOM, which has important consequences for landscape level carbon cycling.

Our findings suggest that microbial diversity apparently remains decoupled from biofilm production as light availability changes. However, the source of the available substrates (i.e. from phototroph exudates or of terrestrial origin) may have modulated microbial diversity along the light gradient. Elevated interactions between biofilm phototrophs and heterotrophs with increasing light availability are also supported by our findings on enzymatic activity. Collectively, our findings therefore evoke that as light becomes attenuated in streams, biofilms rely more on DOM constituents from the streamwater with no apparent impact on microbial diversity but with clear shifts in bacterial community composition and function.

## Experimental procedures

### Microcosm setup and sampling design

Benthic biofilms were established for a total of 27 days on glass slides (1 cm^2^) in streamside flumes fed with raw streamwater from Oberer Seebach (OSB, Lunz am See, Austria, 600 m above sea level) in June 2012 (see SI Methods for further information on OSB). After 18 days of establishment, all slides were brushed with a soft brush to remove loosely attached biomass, and biofilms were then allowed to regrow during 9 days. Based on previous experience (Tom Battin, unpublished), this method yields uniformly compact biofilms that do not easily disintegrate during handling. To achieve a gradient in phototrophic biomass, slides were covered with neutral‐density photographic filters (LEE filters, Burbank, California, USA) during biofilm establishment, generating a light gradient with six levels. These filters attenuate incident light but do not change the spectral distribution and have been previously used for stream biofilm growth (Ceola *et al*., 2013). We used filters 226, 298, 209, 210, 211 and 299 to yield transmission of 92%, 69%, 51%, 24%, 14% and 7% of the incident PAR respectively. Ninety slides were then transferred into each air‐tight Plexiglas© microcosms covered with the same light filters. Each light treatment was replicated in five microcosms. All 30 microcosms were randomly distributed under a fluorescent light source (Phillips, TL‐D 58W/33) with a 14:10 h day : night regime. PAR intensity was measured inside each microcosm (LI‐COR 1400, WALZ US‐SQS/L sensor). Microcosms had magnetic stirring to ensure continuous mixing of water overlying biofilms. Temperature was measured inside selected microcosms and averaged 18.0 ± 0.9°C (mean ± SD) and 16.7 ± 0.5°C during day and night respectively.

Biofilms in the microcosms were incubated with oligotrophic groundwater from a nearby spring, as it is similar to the groundwater that feeds OSB at baseflow. Water in each microcosm was replaced twice daily and spiked with a cold‐water extract from crack willow that served as allochthonous DOM (Wagner *et al*., 2014). This groundwater–willow extract mixture is henceforward referred to as feed water. We collected water samples for the analyses of DOC, P‐PO_4_ and N‐NO_3_ concentration from the feed water and after recirculation (7 h) from the microcosms. Concentrations of DOC, P‐PO_4_ and N‐NO_3_ in the feed water were 0.879 ± 0.053 mg C l^−1^, 69.3 ± 3.8 μg P‐PO_4_ l^−1^ and 1208 ± 17.2 μg N‐NO_3_ l^−1^ respectively. Replicate biofilm samples for 454‐pyrosequencing (3 slides per microcosm), enzyme activity analysis (1 slide per microcosm and enzyme), microbial cell counts (6 slides per microcosm) and the determination of chlorophyll *a* concentration (6 slides per microcosm) were randomly collected from the microcosms at day 1, several hours after the transfer of the biofilms to the microcosms, and at day 7, before the termination of the experiment. These sampling times were chosen to be representative of the experimental period.

### Solute removal and metabolism

The areal removal rates *(R)* of DOC, PO_4_ and NO_3_ were calculated according to R = (ΔC * V)/(T * A) where Δ*C* is the difference in concentration between the feed water and the output of the microcosm measured over one recirculation period, *V* is the water volume (0.75 l) in the microcosm, *T* is the recirculation time (in hours), and *A* is the total surface area (in cm^2^) of all slides present in the microcosm at a given time.

Concentration of dissolved oxygen (DO) was measured at the beginning and at the end of each recirculation period using planar optodes (PSt3 sensor, Presens, Germany). DO production (during day) and DO consumption (during night) were used to infer NPP, R and GPP (Bott, 1983). We assumed a respiration quotient of 0.81 as used previously for freshwater ecosystems (Berggren *et al*., 2011; Bengtsson *et al*., 2014). N‐NO_3_ and P‐PO_4_ concentrations were determined on a continuous flow analyser (FlowSys 3rd generation, SYSTEA Analytical Technologies) on sterile filtered (0.2 μm) samples; DOC concentrations in filtered (Whatmann GFF) samples were measured on a TOC Analyzer (Sievers 5310C, GE Analytical Instruments). All glassware was acid‐washed and combusted (450°C, 4 h).

### Extracellular enzyme activities

Biofilms were sampled for the analysis of extracellular enzyme activity at day 1 and at day 7 of the experiment. The extracellular enzymes leucine‐aminopeptidase (EC 3.4.11.1), phosphatase (EC 3.1.3) and beta‐D‐glucosidase (EC 3.2.1.21) were selected because of their relevance in C‐, N‐ and P‐cycling; their activities were measured spectrofluorometrically using aminomethylcoumarin (AMC) and methylumbelliferyl (MUF) (Sigma Chemical Company). Hydroximethylether was added to the MUF substrates (final solution in the assay of 0.1 %) to facilitate their dissolution in water. The MUF and AMC reference standards were prepared with autoclaved MQ water. Phenol oxidase activity (EC 1.14.18.1) was measured using 3,4‐dihydroxyphenylalanine (l‐DOPA) (Sinsabaugh, 1994). Saturation curves for each enzyme were made before the start of the experiment to determine enzyme–substrate saturation conditions. One biofilm sample per microcosm was collected at day 1 and at day 7 for each extracellular enzyme analysis (*n* = 30 per enzyme), placed into a pre‐combusted glass vial containing 4 ml of sterile filtered (0.2 μM) water from the respective microcosm and the corresponding substrate was added. All assays were conducted under substrate saturation conditions and incubated for 1 h (18°C) on a shaker in the dark to avoid possible photo‐degradation of the substrates. For each enzyme, we used negative controls for substrate color (0.2‐μm‐filtered water from the microcosms and substrate analog) to assess the abiotic degradation of the artificial substrate. At the end of the incubation, glycine buffer (pH = 10.4) was added to the biofilm samples and to the controls. The fluorescence of the MUF‐substrates was read at EX365nm and EM455nm, the fluorescence of the AMC‐substrates was read at EX364nm and EM445nm (F‐7000 Hitachi). Phenol oxidase activity was measured at 450 nm (UV‐1700 PharmaSpec, Shimadzu). Enzyme activities were expressed as nmol of substrate converted h^−1^ cm^−2^.

### DNA extraction, PCR and 454‐pyrosequencing

Biofilms were sampled for 454‐pyrosequencing of the 16S rRNA gene at day 1 and at day 7 of the experiment. Triplicate samples were collected from each microcosm, flash frozen in liquid N_2_ and stored at −80°C. Total nucleic acids were extracted from the biofilm (Urich *et al*., 2008). The bacterial hypervariable regions V3 and V4 of the 16S rRNA gene were amplified with the forward primer 341F (5′‐CTACGGGNGGCWGCAG‐3′) and the reverse primer 805R (5′‐GACTACHVGGGTATCTAATCC‐3′) combination (Logue *et al*., 2012) in a two‐step PCR protocol (for detailed information please see SI Methods). PCR products were purified by agarose gel electrophoresis and the QIAquick Gel Extraction Kit (QIAGEN) following the producer's recommendations. The purified PCR products were quantified using the DNA binding QuantiFluor™ dsDNA System Kit (Promega Corporation). Amplicons were pooled in equimolar concentrations to obtain similar numbers of 454‐pyrosequencing reads per sample. Amplicons were sequenced on a GS FLX Titanium Sequencer at the Center for Genomic Research (University of Liverpool, UK). 454‐Pyrosequencing data were de‐noised and reads were clustered at a 97% identity level to operational taxonomic units (97% OTUs) using the software package AmpliconNoise V1.28 (Quince *et al*., 2011). Taxonomic assignments were determined using CREST (Lanzén *et al*., 2012).

### Microbial cell counts, chlorophyll *a* and bulk biomass

Six biofilm samples were randomly collected from each microcosm at day 1 and at day 7 and conserved in 2.5% formaldehyde (24 h, 4°C). Prior to cell counting, we added 20 ml pyrophosphate (0.025 mM pyrophospate, 2.5% formaldehyde), and samples were shaken (60 min) and sonicated three times for 20 s (14% amplitude, 1s pulse, 1s pause) to disaggregate cells. Larger particles were allowed to settle from the supernatant for 20 min. Nucleic acids were stained using SYTOX Green (Life Technologies Corporation) (5 μM final concentration, 15 min) and cells were counted on a Cell Lab Quanta SC (Beckman Coulter). An additional six glass slides were sampled from each microcosm at day 1 and at day 7 and chlorophyll *a*, the most important photosynthetic pigment in algae and cyanobacteria and a proxy for phototrophic biomass, was extracted in acetone overnight (4°C). Samples were then vortexed, filtered (Whatmann GFF) and absorbance was measured at 665 nm and 750 nm with a spectrophotometer (UV‐1700 PharmaSpec, Shimadzu). Bulk biomass was measured as C content of six biofilm samples using an Elemental Analyzer (EA1110; CE Instruments, Thermo Fisher).

### Data analyses

For nMDS ordination, phototrophic‐ (*Cyanobacteria* and algal plastid), unclassified‐ and rare 97% OTUs (present in less than 5% of the samples) were excluded. We computed a similarity matrix using the Bray–Curtis index and subjected it to nMDS to visualize the community dynamics of the biofilms. PERMANOVA was used to test the significant effect of light intensity, GPP and time on the bacterial community composition. Alpha diversity was calculated as 97% OTU richness, evenness, the number equivalents of the Shannon and of the Gini–Simpson index from resampled 454‐pyrosequencing data, excluding 97% OTUs identified as *Cyanobacteria* and algal plastids (Jost *et al*., 2010; Besemer *et al*., 2013). We used this family of indices as they differently weigh abundant and rare species. All samples were rarefied to the lowest number of reads (*n* = 1002) obtained from a sample prior to analysis. Taxon relative abundance, alpha diversity and biofilm biomass measures were tested for significant differences between light treatments using ANOVA and post hoc Tukey test. We recognize the limitations inherent to comparison of relative abundances, as a change in the relative abundance of a taxon may not reflect a corresponding change in absolute abundances. Nonetheless, relative abundances as are produced by sequencing approaches are frequently used to characterize changes in overall microbial community compositions (e.g. Gilbert *et al*., 2011).

We used the extracellular enzyme activities to infer a multifunctionality index (Gamfeldt *et al*., 2008). Multifunctionality was calculated using the individual extracellular enzyme activities from day 1 and from day 7 of the experiment. If one of the enzyme activities dropped below a pre‐defined threshold (0.5, 0.75, 0.9) of the maximal enzyme activity for this enzyme, the function was considered lost. This means for the 0.5 threshold, for instance, that as long as the community is able to perform 50% of the maximal enzyme activity in all samples, the function is considered retained, whereas if functioning for a specific enzyme dropped below 50% a loss of function was inferred. Spearman's rank correlation was used to test significant correlations between species richness and evenness, respectively, and GPP. All statistical analyses were performed using the software and statistical computing environment R (R Development Core Team, 2014).

## Supporting information


**Table S1.** Biofilm parameters from all light treatments (relative transmission (%T) of the incident light) at day 1 and at day 7 of the experiment; given are mean ± SD over the light treatments; analysis of variance (ANOVA) displays significant differences (*P* < 0.05) between light treatments.
**Table S2.** Alpha diversity of the biofilm community from all light treatments (relative transmission (%T) of the incident light) at day 1 and at day 7 of the experiment; given are mean ± SD over the light treatments; analysis of variance (ANOVA) displays significant differences (*P* < 0.05) between light treatments.
