# Supplementary material for: Light availability affects stream biofilm bacterial community composition and function, but not diversity
Source: Environ Microbiol. 2015 Jul 22;17(12):5036–47. doi: 10.1111/1462-2920.12913 (PMC4950016; doi:10.1111/1462-2920.12913)
Supplement: Supplementary file 1 — Table S1. Biofilm parameters from all light treatments (relative transmission (%T) of the incident light) at day 1 and at day 7 of the experiment; given are mean ± SD over the light treatments; analysis of variance (ANOVA) displays significant differences (P < 0.05) between light treatments. Table S2. Alpha diversity of the biofilm community from all light treatments (relative transmission (%T) of the incident light) at day 1 and at day 7 of the experiment; given are mean ± SD over the light treatments; analysis of variance (ANOVA) displays significant differences (P < 0.05) between light treatments. [file EMI-17-5036-s001.zip › EMI_12913-supp-0001-SI_methods.docx]

**Supplementary methods**

**Experimental conditions.**

Some conditions differed between the outdoor streamside flumes where biofilms were established and the microcosms where they were kept during the experiment. For example, temperature averaged 18.0±0.9 °C during day and 16.7±0.5 °C during night in the microcosms whereas the average temperature in the flume was 9.3±1.6 °C. Light conditions also differed, with a maximum of 152 μmol photons s^-1^ m^-1^ achieved by the fluorescent light source, while outdoor daylight conditions typically range between 150 and 2000 μmol photons s^-1^ m^-1^ in the area (not measured during biofilm establishment). DOC concentrations were of a similar range in the microcosms (feed water mean: 0.879±0.053 mg L^-1^ recirculated mean: 1.14±0.13 mg L^-1^) compared to the typical ambient concentrations encountered in the OSB. Likewise, NO_3_ concentrations were not unusual compared to natural conditions. However, the concentration of PO_4_ was higher in the microcosms (feed water mean: 69.3±3.8 µg L^-^1) than what is typical for the OSB (<10 µg L^-^1).

**Primer specifications and PCR conditions.**

First, the universal forward primer 341F (5’-CTACGGGNGGCWGCAG-3’) and reverse primer 805R (5'-GACTACHVGGGTATCTAATCC-3') (Thermo Fisher Scientific Inc.) were used to amplify the bacterial genomic DNA. Second, the products from the first PCR were amplified with 341F and 805R amended with the 454-Titanium A and B adaptors, respectively. In addition, the forward primer contained unique barcodes. DNA concentrations of the samples were determined (QuantiFluor™ dsDNA System, Promega Corporation) and template input was adjusted to equal concentrations in all PCR reactions. The 20 µl PCR reactions contained each primer without barcodes at 0.4 mmol L^-1^ (Thermo Fisher Scientific Inc.), dNTPS at 0.65 mmol L^-1^ (Thermo Fisher Scientific Inc.), bovine serum albumin at 40 mg L^-1^ (Thermo Fisher Scientific Inc.), MgCl_2_ at 2.0 mmol L^-1^ (Thermo Fisher Scientific Inc.) and Phusion High-Fidelity DNA Polymerase (2U µl^-1^) with the recommended PCR buffer (Thermo Fisher Scientific Inc.).

In the first PCR step, the 16S rRNA gene was amplified using an initial denaturing step at 94°C for 1 min, followed by 25 cycles of denaturation at 94°C for 30s, annealing for 30s (touchdown PCR; starting at 62°C and decreasing to 50°C), elongation at 72°C for 1 min and a final elongation at 72°C for 10 min. Each reaction was run in technical duplicates and subsequently pooled. In the second PCR step, the conditions were modified slightly: Bovine serum albumin was omitted, primers with adaptors and barcodes at concentrations of 0.8 mmol l^-1^ were used. The annealing temperature was fixed at 56°C for 30 s and the PCR was run for only 5 cycles. The following accession number was obtained upon submission of the sequences: SRX803716.
