# Supplementary material for: Light availability affects stream biofilm bacterial community composition and function, but not diversity
Source: Environ Microbiol. 2015 Jul 22;17(12):5036–47. doi: 10.1111/1462-2920.12913 (PMC4950016; doi:10.1111/1462-2920.12913)
Supplement: Supplementary file 1 — Table S1. Biofilm parameters from all light treatments (relative transmission (%T) of the incident light) at day 1 and at day 7 of the experiment; given are mean ± SD over the light treatments; analysis of variance (ANOVA) displays significant differences (P < 0.05) between light treatments. Table S2. Alpha diversity of the biofilm community from all light treatments (relative transmission (%T) of the incident light) at day 1 and at day 7 of the experiment; given are mean ± SD over the light treatments; analysis of variance (ANOVA) displays significant differences (P < 0.05) between light treatments. [file EMI-17-5036-s001.zip › EMI_12913-supp-0002-SI_tables.docx]

|  |  | 92%T  (a) | 69%T  (b) | 51%T  (c) | 24%T  (d) | 14%T  (e) | 7%T  (f) | ANOVA |
| --- | --- | --- | --- | --- | --- | --- | --- | --- |
| Day 1 | Chlorophyll *a* (µg cm^-2^) | 2.1±1.0 | 2.1±0.9 | 2.4±0.8 | 3.1±0.8 | 2.8±0.9 | 0.9±0.5 | d-f, e-f |
|  | Biomass (mg C cm^-2^) | 0.37±0.32 | 0.60±0.55 | 0.23±0.12 | 0.29±0.20 | 0.15±0.11 | 0.11±0.04 |  |
|  | Cell count (10^7^ cm^-2^) | 3.1±1.9 | 3.8±1.5 | 4.1±1.9 | 3.8±1.4 | 2.7±1.1 | 1.8±0.5 |  |
|  | Net primary production (µg C h^-1^ cm^-2^) | 3.0±0.8 | 3.0±0.2 | 2.6±0.7 | 2.5±0.3 | 1.6±0.6 | 0.9±0.5 | a-e, b-e, a-f, b-f, c-f, d-f |
| Day 7 | Chlorophyll *a* (µg cm^-2^) | 3.8±0.7 | 3.7±0.7 | 3.7±0.4 | 4.0±0.4 | 3.2±1.1 | 2.0±0.5 | a-f, b-f, c-f, d-f |
|  | Biomass (mg C cm^-2^) | 0.37±0.13 | 0.42±0.12 | 0.21±0.03 | 0.31±0.22 | 0.13±0.06 | 0.06±0.03 | a-e, a-f, b-e, b-f , d-f |
|  | Cell count (10^7^ cm^-2^) | 9.0±1.2 | 10.1±2.6 | 8.0±2.7 | 7.9±2.1 | 6.0±0.9 | 3.4±1.1 | b-e, a-f, b-f, c-f, d-f, e-f |
|  | Net primary production (µg C h^-1^ cm^-2^) | 7.9±0.8 | 7.9±1.7 | 5.8±1.1 | 4.3±0.8 | 2.3±0.3 | 1.6±0.3 | a-c, a-d, a-e, a-f, b-c, b-d, b-e, b-f, c-e, c-f, d-e, d-f |

**Table S1.** Biofilm parameters from all light treatments (relative transmission (%T) of the incident light) at day 1 and at day 7 of the experiment; given are mean ± SD over the light treatments; analysis of variance (ANOVA) displays significant differences (*P* < 0.05) between light treatments.

**Table S2.** Alpha diversity of the biofilm community from all light treatments (relative transmission (%T) of the incident light) at day 1 and at day 7 of the experiment; given are mean ± SD over the light treatments; analysis of variance (ANOVA) displays significant differences (*P* < 0.05) between light treatments.

|  |  | 92%T  (a) | 69%T  (b) | 51%T  (c) | 24%T  (d) | 14%T  (e) | 7%T  (f) | ANOVA |
| --- | --- | --- | --- | --- | --- | --- | --- | --- |
| Day 1 | Richness | 414.9±20.3 | 430.7±23.7 | 448.8±10.3 | 476.7±33.6 | 415.8±31.8 | 418.5±49.3 | a-d, d-e |
|  | Simpson NE | 130.7±15.9 | 138.3±16.8 | 153.9±6.6 | 181.8±29.7 | 121.1±38.3 | 131.3±28.7 | a-d, d-e, d-f |
|  | Shannon  NE | 240.4±21.7 | 254.1±23.2 | 275.5±8.4 | 309.7±35.6 | 237.4±41.3 | 243.9±45.2 | a-d, e-d, e-f |
|  | Evenness | 0.91±0.01 | 0.91±0.01 | 0.92±0.003 | 0.93±0.01 | 0.91±0.02 | 0.91±0.01 | d-e |
| Day 7 | Richness | 370.6±59.0 | 361.6±42.5 | 371.2±64.2 | 399.1±31.7 | 346.7±54.5 | 379.4±43.4 |  |
|  | Simpson NE | 119.3±44.9 | 103.1±19.6 | 100.0±52.7 | 113.8±32.2 | 90.6±31.6 | 118.2±28.8 |  |
|  | Shannon NE | 210.8±59.9 | 196.0±34.3 | 197.9±71.9 | 225.9±35.6 | 183.0±48.3 | 216.5±42.4 |  |
|  | Evenness | 0.90±0.02 | 0.89±0.01 | 0.90±0.02 | 0.90±0.02 | 0.89±0.02 | 0.90±0.02 |  |
